# Impact of phosphoinositide-3-kinase and vitamin D3 nuclear receptor single-nucleotide polymorphisms on the outcome of malignant melanoma patients

**DOI:** 10.18632/oncotarget.18304

**Published:** 2017-05-30

**Authors:** Francesca Morgese, Davide Soldato, Silvia Pagliaretta, Riccardo Giampieri, Donatella Brancorsini, Mariangela Torniai, Silvia Rinaldi, Agnese Savini, Azzurra Onofri, Marina Scarpelli, Rossana Berardi

**Affiliations:** ^1^ Clinica Oncologica, Università Politecnica delle Marche, Azienda Ospedaliero-Universitaria Ospedali Riuniti “Umberto I°-G.M. Lancisi-G. Salesi”, Ancona, Italy; ^2^ Section of Pathological Anatomy and Histopathology, Deparment of Neuroscience, Università Politecnica delle Marche, Azienda Ospedaliero-Universitaria Ospedali Riuniti “Umberto I°-G.M. Lancisi-G. Salesi”, Ancona, Italy

**Keywords:** PI3K SNPs, VDR SNPs, allele frequency, survival, melanoma

## Abstract

**Background:**

Several studies associating single nucleotide polymorphisms (SNPs) frequencies with tumors outcome have been conducted, nevertheless malignant melanoma literature data are inconclusive.

Therefore we evaluate the impact of different genotypes for phosphoinositide-3-kinase (PI3K) and vitamin D3 nuclear receptor (VDR) SNPs on melanoma patients’ outcome.

**Materials and methods:**

Genomic DNA of 88 patients was extracted from blood and tumor samples. SNPs were determined by PCR using TaqMan assays. We selected polymorphisms of the regulatory and catalytic subunit of PI3K (PIK3R1 and PIK3CA genes, respectively), analyzing rs2699887C>T of *PIK3CA* and rs3730089G>A of *PIK3R1* SNPs. Furthermore we considered the following *VDR* SNPs: rs2228570A>G (Fok1), rs731236A>G (Taq1) and rs1544410C>T (Bsm1).

Progression free survival (PFS) and overall survival (OS) were estimated with the Kaplan-Meier method and with Mantel-Haenszel log-rank test.

**Results:**

The statistical analysis for Fok1 of *VDR* showed a significant difference in PFS after the first line therapy (median PFS= 21.2 months in the homozygous recessive genotype group vs. 3.3 months of homozygous dominant and heterozygous ones, *p*= 0.03). In particular, in homozygous recessive patients for Fok1 SNPs of *VDR* a high rate of histological regression and BRAF (B- Rapidly Accelerated Fibrosarcoma gene) mutation were observed. Furthermore, more efficacy of BRAF +/- MEK (MAPK-ERK-Kinase) inhibitors therapies in homozygous recessive patients vs. homozygous dominant and heterozygous ones was shown.

**Conclusions:**

Our study showed a significant correlation between homozygous recessive genotype of Fok1 SNPs of VDR gene and an increased PFS in patients who underwent a first line therapy with BRAF inhibitors.

## INTRODUCTION

Malignant melanoma is the most aggressive skin cancer and the fifth and seventh most common cancer in developed countries among men and women, respectively. By the end of 2016 it is estimated that 76,380 malignant melanoma of the skin will be diagnosed and 10,130 disease-related deaths will occur, in the United States only [1].

The 5-year probability of surviving malignant melanoma ranges from 98% to <20% [2] depending on the stage of disease at diagnosis [3].

Evidences suggest that sex, age at presentation, histological features and stage of disease at time of diagnosis represent important factors influencing melanoma progression and survival [4–15].

Recently, important biological mechanisms have been examined for their contribution to the development and progression of malignant melanoma, including the phosphatidylinositol 3-kinase (PI3K)/PTEN/AKT/mTOR signaling pathway and the nuclear vitamin D receptor (VDR) function.

Many growth factors and cytokines are responsible for the activation of these two pathways implicating cell growth and death [16, 17].

In particular, numerous and different human tumors exhibited genetic alterations in the PI3K pathway. Specifically, *PIK3CA*, which encodes for the catalytic subunit p110α of class IA PI3-kinase, is amplified and overexpressed in ovarian cancer [18] and is commonly gain-of-function mutations in colon [19], brain [20], breast [21] and gastric cancers [22–25], but *PIK3CA* mutations seem to have a limited impact on growth of melanoma, up to now [26].

On the other side *PIK3R1*, encoding for the regulatory subunit p85 of PI3K, shows mutations especially in ovarian, colon [27], endometrial [28], prostate cancer [29], glioblastoma [30] and also in malignant melanoma [31].

Finally, considering VDR, its higher expression has been related to better survival in patients with lung [32, 33] and breast cancer [34, 35].

A lower VDR expression, at the same time, was observed in melanoma especially for the vertical growth phase versus normal skin or nevi [36] and hypo-activation of VDR signaling pathway can inhibit melanocytic progression [37].

Frequent epigenetic and genetic alterations, such as single-nucleotide polymorphisms (SNPs), may increase or decrease the function of genes in VDR signaling pathways, resulting in susceptibility and modified prognosis of malignant melanoma [38–40].

Some variants of *PIK3CA* and *PIK3R1* SNPs were studied in different type of tumor demonstrating an influence on prognosis [41–47] but in malignant melanoma, PI3K polymorphisms have not been evaluated yet.

For more common variants of polymorphisms in the VDR gene, available data indicated their capacity to change disease-specific survival in patients with breast cancer [48], lung cancer [49, 50] ovarian cancer [51] colorectal cancer [52, 53], renal cell carcinoma [54], prostate tumor [55–57], head and neck squamous cell carcinoma [58, 59], glioma [60] and, only very recently, in patients with malignant melanoma [46].

Currently, the treatment of advanced malignant melanoma made use of immuno-therapy with PD-1 and CTLA-4 inhibitors and, for melanomas harboring BRAF mutation, also, of target therapy with BRAF and MEK inhibitors.

Our study aimed to examine the associations between the clinical outcomes of malignant melanoma and some variants of SNPs in PIK3CA, PIK3R1 and VDR genes.

## RESULTS

We enrolled 88 consecutive patients treated for cutaneous or occult malignant melanoma between 2012 and 2016: 48 with no evidence of disease (NED) and 40 with metastatic melanoma. Genotypes characteristics of the studied population are summarized in Table 1.

**Table 1:** distribution of polymorphism genotypes among NED and metastatic patients

*Gene*	*Polymorphism*	*Patients’ group^a^*	*Patients in NED*	*Metastatic patients*	*Unknown*
*PIK3CA*	rs2699887	HD	34 (38.6%)	24 (27.2%)	2 (2.3%)
		Ht	11 (12.5%)	13 (14.8%)	
		HR	2 (2.3%)	2 (2.3%)	
*PIK3R1*	rs3730089	HD	38 (43.2%)	33 (37.5%)	1 (1.1%)
		Ht	8 (9.1%)	6 (6.8%)	
		HR	2 (2.3%)	0	
*VDR*	rs2228570	HD	22 (25.0%)	17 (19.3%)	2 (2.3%)
		Ht	20 (22.7%)	16 (18.2%)	
		HR	6 (6.8%)	5 (5.7%)	
	rs731236	HD	16 (18.2%)	17 (19.3%)	4 (4.5%)
		Ht	21 (23.9%)	15 (17.0%)	
		HR	8 (9.1%)	7 (8.0%)	
	rs1544410	HD	18 (20.4%)	17 (19.3%)	2 (2.3%)
		Ht	29 (33.0%)	22 (25.0%)	
		HR	0	0	

^a^HD, Ht and HR stand respectively for: homozygous dominant, heterozygous and homozygous recessive.

We did not observe a statistically significant difference in frequency distribution of polymorphisms between NED patients and metastatic ones.

The MAF (minor allele frequency) for all studied SNPs was >10%, therefore most statistical analysis were conducted applying a dominant model: patients with homozygous dominant and heterozygous genotype were compared with those with homozygous recessive ones.

In the pooled population, homozygous recessive genotype was not detected for SNP rs1544410 (Bsm1) of *VDR* while for SNP rs3730089 of *PIK3R1* the number of homozygous recessive patients was too small to obtain statistical results, therefore we compared homozygous dominant with heterozygous patients.

All the examined polymorphisms fit the Hardy-Weinberg equilibrium (HWE), except for Bsm1 SNP of VDR gene.

Therefore we did not include data about Bsm1.

In terms of OS, we observed no statistical differences between the two groups of patients evaluated for the SNPs rs3730089 in the PIK3R1 gene and *VDR* SNPs rs2228570 (Fok1), rs731236 (Taq1).

**Figure 1:**
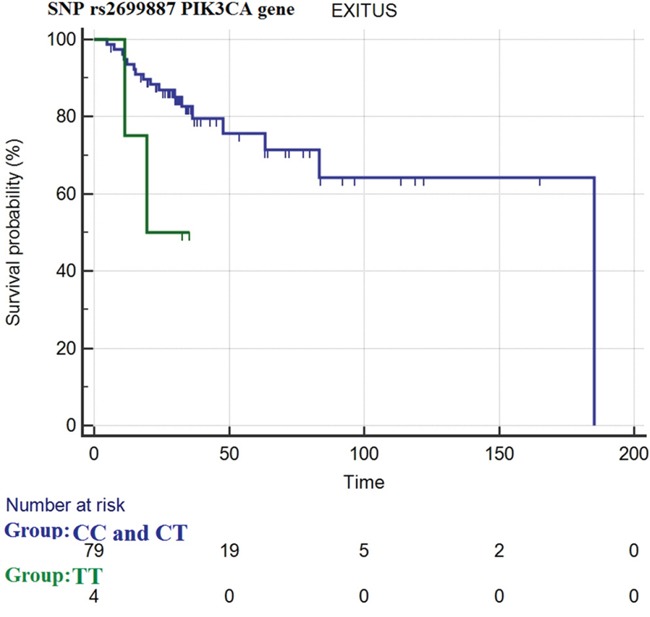
overall survival curves for SNP rs2699887 of PIK3CA gene in homozygous dominant (genotype CC) and heterozygous (genotype CT) patients (blue curve) and homozygous recessive patients (genotype TT) (green curve)

Nevertheless, for the SNP rs2699887 of PIK3CA gene, a very interesting trend of better OS in dominant homozygous and heterozygous genotype vs. recessive homozygous one was shown (median OS= 185.1 months vs. 19.4 months, respectively with a hazard ratio (HR)= 0.28 (95% CI: 0.02-3.61), *p*= 0.06) (Figure 1).

Furthermore, in the cohort of metastatic patients we identified no statistical differences, in terms of PFS, between the selected groups for SNPs rs2699887 in the PIK3CA gene, rs3730089 in the PIK3R1 gene and Taq1 in the VDR gene.

**Figure 2:**
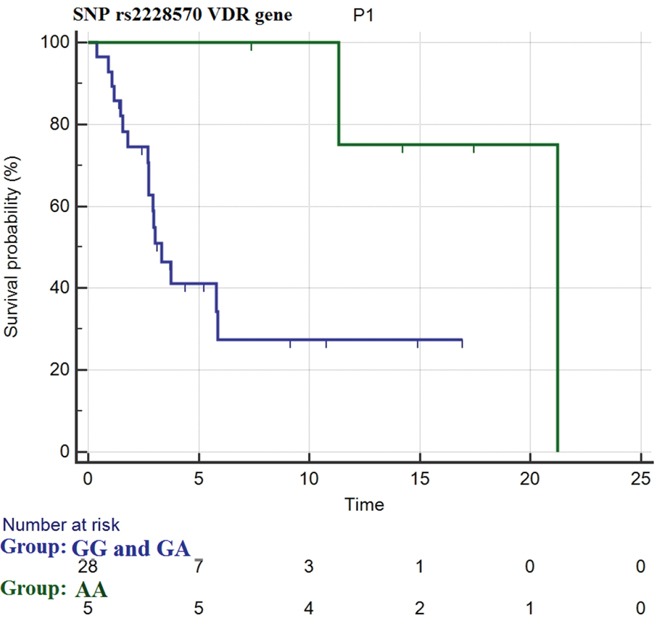
progression free survival for SNP rs2228570 of VDR gene in homozygous dominant (genotype GG) and heterozygous patients (genotype GA) (blue curve) and homozygous recessive patients (genotype AA) (green curve)

Considering the SNP Fok1, we observed statistical differences in PFS after the first line of treatment in favor of homozygous recessive patients vs. homozygous dominant and heterozygous ones (median PFS= 21.2 months vs. 3.3 months, respectively with HR= 0.26 (95% CI: 0.09-0.69), *p*= 0.03 (Figure 2).

In particular, we observed that the homozygous recessive genotype in the Fok1 polymorphism is associated with a higher rate of tumor histological regression (28% vs 8% in homozygous dominant and heterozygous patients; *p*= 0.05) and a higher incidence of BRAF mutations (100% vs 36% in homozygous dominant and heterozygous patients; *p*= 0.01) in the primary melanoma. Among the metastatic patients, 17 of 40 (42.5%) resulted in melanomas harboring BRAF mutation, and 12 of them (70.6%), received BRAF +/- MEK inhibitors as first line of treatment; the treatment of the remaining patients is shown in Table 2. Furthermore, patients carrying a homozygous recessive genotype (41.7%) in the Fok1 polymorphism showed a better disease control rate by the first line of treatment with anti-BRAF +/- anti-MEK drugs vs. homozygous dominant (25.0%) and heterozygous groups (33.3%), with disease stability in 60% of cases vs. 25%, respectively (*p*= 0.01). The complete results of the statistical analysis are shown in Table 3.

**Table 2:** summary of first line treatment in BRAF mutated patients

BRAF mutated patients	
Treatment	N (%)
Chemotherapy	1 (5.9)
BRAF +/- MEK inhibitors	12 (70.6)
ANTI-PD1	1 (5.9)
Best supportive care	3 (17.6)

**Table 3:** hazard ratios (HR) with 95% CI and p value of the Mantiel-Haenszel log-rank test for the studied polymorphisms in the different patients groups

*Gene*	*Polymorphism*	*Patients’ group*	*OS*		*PFS*	
			*HR (95% CI)*	*P value*	*HR*	*P value*
*PIK3CA*	rs2699887	HD and Htvs.HR	0.28 (0.02 – 3.61)	0.0670	0.51 (0.03 – 7.94)	0.503
*PIK3R1*	rs3730089	HDvs.Ht	2.11 (0.78 – 5.66)	0.186	1.71 (0.60 – 4.84)	0.333
*VDR*	rs2228570	HD and Htvs.HR	1.27 (0.33 – 4.84)	0.742	**0.26 (0.09 – 0.69)**	**0.030**
	rs731236	HD and Htvs.HR	1.02 (0.30 – 3.48)	0.964	1.13 (0.39 – 3.29)	0.812
	rs1544410	HDvs.Ht	1.50 (0.62 – 3.62)	0.345	1.35 (0.55 – 3.27)	0.478

## DISCUSSION

The primary endpoint of our study was to assess whether SNPs genotype in key genes associated with tumor regression or progression have an impact on patients prognosis in terms of OS and PFS.

In melanoma patients VDR polymorphisms have been largely studied for their role in the development of the disease [61–65], while their influence on prognosis and survival has been evaluated by a limited number of studies.

The prospective study conducted by Newton-Bishop *et al.* [66] considered 872 patients to identify an association between circulating levels of vitamin D and Breslow thickness; in addiction, five SNPs, including Fok1, Bsm1 and Taq1 were genotyped. Except for a weak association between the risk of relapse and the Bsm1 A allele and low circulating levels of vitamin D, no other impact of the SNPs on disease relapse or overall survival was observed. Annika Shäfer *et al.* [67] evaluated SNPs in VDR gene and other genes involved in vitamin D metabolism in 305 patients with melanoma and 370 healthy volunteers to find a connection between genotype, risk of developing melanoma and its prognosis. No interesting data seemed to arise from this study.

Recently Orlow *et al.* [46] analyzed the genotype of 38 different SNPs in the VDR gene in 3566 patients to assess their role on melanoma specific-survival. Their results showed that a statistically significant association exists between survival and the SNPs Bsm1 and Taq1 also analyzed in our study. Differently from their results, we showed no influence on OS by aforementioned SNPs presumably due to our small sample and short median time of follow-up (2.6 years compared to 7.6 years of Orlow *et al.*'s study).

To the best of our knowledge this is the first study which aimed to evaluate the influence of SNPs in the VDR gene on response to antitumor treatment, demonstrating that homozygous recessive patients for Fok1 polymorphism have a longer progression free survival and a better disease control rate to the first line of treatment with anti-BRAF +/- anti-MEK drugs when compared to homozygous dominant and heterozygous genotypes.

Nowadays limited data have been published about the SNP rs2699887 in the PIK3CA gene and it has not been investigated in melanoma patients so far.

In particular the aforementioned SNP was studied in esophageal [68], endometrial [28], oral [69], pulmonary [70, 71], and colorectal cancer [72] with various endpoints: risk of tumor development, association with pathological features, risk of metastasis, toxicity to chemotherapy and survival.

Wang *et al.* [45] studied 115 patients with endometrial cancer observing a better survival for heterozygous genotype in the PIK3CA rs2699887.

In our study we identified a trend (*p*= 0.06) of worse OS in recessive homozygous patients for PIK3CA rs2699887 SNPs vs. dominant and heterozygous ones. This SNPs could become a very interesting prognostic biomarker if it will be confirmed and demonstrated significative in future research.

Few data has been published on the SNP rs3730089 in the PIK3R1 gene, studied only in prostate [73] and colon cancer. Despite no association between the SNP and prostate cancer risk was observed, the homozygous recessive and heterozygous genotypes have been associated with a greater risk of developing colon cancer [74].

Our study is the first to evaluate this SNP rs3730089 in the PIK3R1 gene in melanoma patients and its impact on prognosis, even though no statistical association was found between genotype, OS and PFS.

In conclusion, in our study we showed for the first time that homozygous recessive patients for Fok1 polymorphism have a longer progression free survival and better disease control rate during the first line of treatment with anti-BRAF +/- anti-MEK drugs compared to homozygous dominant and heterozygous genotypes. Furthermore, we first studied SNPs rs3730089 in the PIK3R1 gene and rs2699887 in the PIK3CA gene in melanoma patients, observing a trend of worse OS in homozygous recessive patients for the SNP rs2699887 in the PIK3CA gene vs. homozygous dominant and heterozygous genotypes.

The main study limitation is related to the small sample of patients: increasing sample size and time of follow-up would improve the statistical power of our findings allowing us to confirm, most likely, the prognostic and predictive role of the SNPs Fok1 in the VDR gene and rs2699887 in the PIK3CA gene. There are no evidence-based guidelines, up to now, that specify the best therapy sequence for treatment of metastatic melanoma (immunotherapy followed by target treatments or vice versa), therefore it could be interesting to evaluate whether the Fok1 polymorphism is associated with a major efficacy of treatment with BRAF +/- MEK inhibitors regardless of line of therapy.

## MATERIALS AND METHODS

### Patients’ selection

We retrospectively reviewed clinical history and follow-up of patients with cutaneous or occult malignant melanoma treated at the Department of Medical Oncology and at the Dermatology Unit of Università Politecnica Marche-Azienda Ospedaliero Universitaria Ospedali Riuniti Umberto I°-G.M. Lancisi-G. Salesi in Ancona, Italy between 2012 and 2016.

Eligibility criteria included histological diagnosis of malignant melanoma and no contraindications to anti-tumor therapy. Data was retrospectively collected from patients’ medical records.

Recorded patient characteristics and clinical-pathological features included: age, sex, Eastern Cooperative Oncology Group (ECOG) performance status, stage of disease according to the TNM (AJCC 2009) [3], site of tumor, histological features, mutational status of BRAF gene, allele frequencies of the analyzed SNPs and data regarding all the treatments performed by the patients.

For patients who underwent immunotherapy, response to treatment was evaluated with the immune related Response Criteria (irRC) [75].

For all the others, response to therapy was assessed according to RECIST 1.1 (Response Evaluation Criteria In Solid Tumors) [76]. The “Common Terminology Criteria for Adverse Events” (NCI CTCAE, version 4.0) [77] was used to record side effects of anti-tumor therapy.

### PI3K and VDR genotyping

DNA extracted from formalin-fixed paraffin-embedded tissue blocks of malignant melanoma or from whole blood was used to PIK3CA, PIK3R1 and VDR genotyping.

Single nucleotide polymorphisms within each gene were selected using the Single Nucleotide Polymorphism database (dbSNP) generated by the National Centre for Biotechnology Information [78] and by reviewing the published literature, using the following criteria:

1. the polymorphism had some degree of likelihood to biologically modify the structure or the expression of the gene.
2. the MAF was above 10%.
3. the genetic polymorphism was well-documented.

On the basis of aforementioned criteria, we selected the following SNPs:

- PIK3CA gene: rs2699887 SNP, located in an intron region in proximity of the 5'-side, with C/T allelic variants.
- PIK3R1 gene: rs3730089 missense SNP, located in exon 6, with A/G allelic variants. In presence of minor allelic variant, PI3K/AKT pathway becomes hyperactivated [79].
- VDR gene: rs2228570 missense SNP, named Fok1, located near starting codon, with T/C variants that defined f and F alleles respectively [80, 81].
- VDR gene: rs731236 SNP, named Taq1, located in exon 2, with C/T variants that define t and T alleles respectively.
- VDR gene: rs1544410 SNP, named Bsm1, located in an intron region, with A/G variants [82].

SNP genotyping was performed by TaqMan technology, using SNP genotyping assay. Genotypes were analysed on the 7300 Real-Time PCR System.

### Statistical analysis

The association between categorical variables was evaluated by Fisher exact test for binomial categorical variables and by chi-square test for all other applications. Survival probability was estimated using the Kaplan–Meier method.

Significant differences in the probability of survival between the strata were evaluated by log-rank test (significance was set at a 0.05 level for all analyses).

For statistical analysis, OS was evaluated from histological diagnosis of melanoma to event or censoring, whereas PFS was considered as the interval between the date of start of 1 st line treatment until death, last follow-up visit or first sign of clinical progression, whichever came first.

Statistical analysis for polymorphisms was conducted considering the outcome of wild type (homozygous dominant) and heterozygous vs. homozygous recessive genotype.

Nevertheless, in the pooled population, two polymorphisms showed the absence or a poorly representation of homozygous recessive genotype. In these cases for the statistical analysis we confronted wild type with heterozygous patients.

All genetic polymorphisms were examined for deviation from HWE using the Powermarker v.3.25 package [83]. All statistical analyses were performed by using MedCalc Statistical Software version 14.10.2 [84].

### Ethic statement

Ethical committee of our Institution (Comitato Etico Regionale delle Marche, Azienda Ospedaliero-Universitaria Ospedali Riuniti Ancona, Via Conca 71, 60126, Ancona, Italy) accepted this study.

All patients accorded their written consent to the research and to all the diagnostic-therapeutic procedures.

All authors declare that have not received fees for serving as a speakers or consultants and/or an advisory board members for any organizations. All authors have no received research funding from any organizations. No authors are employees of any organization. No authors own stocks and/or shares in organization. No authors own patent.

All authors declare that they have no competing interests.

All authors contributed to the editorial, read and approved the final manuscript.

Disclose any potential conflicts of interest.
